# Attenuation of the Infiltration of Angiotensin II Expressing CD3^+^ T-Cells and the Modulation of Nerve Growth Factor in Lumbar Dorsal Root Ganglia – A Possible Mechanism Underpinning Analgesia Produced by EMA300, An Angiotensin II Type 2 (AT_2_) Receptor Antagonist

**DOI:** 10.3389/fnmol.2017.00389

**Published:** 2017-11-21

**Authors:** Nemat Khan, Arjun Muralidharan, Maree T. Smith

**Affiliations:** ^1^UQ Center for Clinical Research, Faculty of Medicine, The University of Queensland, Brisbane, QLD, Australia; ^2^School of Pharmacy, Faculty of Health and Behavioural Sciences, The University of Queensland, Brisbane, QLD, Australia

**Keywords:** allodynia, analgesia, angiotensin II, AT_2_ receptor, AT_2_ receptor antagonist, CCI-rat model, nerve growth factor, peripheral neuropathic pain

## Abstract

Recent preclinical and proof-of-concept clinical studies have shown promising analgesic efficacy of selective small molecule angiotensin II type 2 (AT_2_) receptor antagonists in the alleviation of peripheral neuropathic pain. However, their cellular and molecular mechanism of action requires further investigation. To address this issue, groups of adult male Sprague–Dawley rats with fully developed unilateral hindpaw hypersensitivity, following chronic constriction injury (CCI) of the sciatic nerve, received a single intraperitoneal bolus dose of the small molecule AT_2_ receptor antagonist, EMA300 (10 mg kg^-1^), or vehicle. At the time of peak EMA300-mediated analgesia (∼1 h post-dosing), groups of CCI-rats administered either EMA300 or vehicle were euthanized. A separate group of rats that underwent sham surgery were also included. The lumbar (L4–L6) dorsal root ganglia (DRGs) were obtained from all experimental cohorts and processed for immunohistochemistry and western blot studies. In vehicle treated CCI-rats, there was a significant increase in the expression levels of angiotensin II (Ang II), but not the AT_2_ receptor, in the ipsilateral lumbar DRGs. The elevated levels of Ang II in the ipsilateral lumbar DRGs of CCI-rats were at least in part contributed by CD3^+^ T-cells, satellite glial cells (SGCs) and subsets of neurons. Our findings suggest that the analgesic effect of EMA300 in CCI-rats involves multimodal actions that appear to be mediated at least in part by a significant reduction in the otherwise increased expression levels of Ang II as well as the number of Ang II-expressing CD3^+^ T-cells in the ipsilateral lumbar DRGs of CCI-rats. Additionally, the acute anti-allodynic effects of EMA300 in CCI-rats were accompanied by rescue of the otherwise decreased expression of mature nerve growth factor (NGF) in the ipsilateral lumbar DRGs of CCI-rats. In contrast, the increased expression levels of TrkA and glial fibrillary acidic protein in the ipsilateral lumbar DRGs of vehicle-treated CCI-rats were not attenuated by a single bolus dose of EMA300. Consistent with our previous findings, there was also a significant decrease in the augmented levels of the downstream mediators of Ang II/AT_2_ receptor signaling, i.e., phosphorylated-p38 mitogen-activated protein kinase (MAPK) and phosphorylated-p44/p42 MAPK, in the ipsilateral lumbar DRGs.

## Introduction

Peripheral neuropathic pain may develop as a consequence of nerve injury or disease affecting the peripheral components of the somatosensory nervous system (Finnerup et al., 2015). This type of chronic pain often remains poorly alleviated by clinically available analgesics and/or adjuvants recommended by the Neuropathic Pain Special Interest Group of the International Association for the Study of Pain, due to lack of efficacy and/or dose-limiting adverse effects (Finnerup et al., 2015). Poorly alleviated peripheral neuropathic pain adversely affects quality of life and imposes an enormous economic burden on patients, their families and care-givers as well as society (Finnerup et al., 2015). Over the past two decades, numerous ‘pain targets’ have been discovered (Sisignano et al., 2016; Bannister et al., 2017). However, despite robust preclinical findings, most of these candidate analgesic and adjuvant drugs have failed to show promising analgesic efficacy and/or an acceptable tolerability profile in subsequent proof-of-concept clinical trials in humans (Woolf, 2010; Oertel and Lotsch, 2013).

A notable exception is the small molecule angiotensin II type 2 (AT_2_) receptor antagonists (e.g., EMA200, EMA300 and EMA401) that exhibit >1000-fold binding selectivity over the angiotensin II type 1 (AT_1_) receptor (Smith et al., 2013a, 2016). To date, a number of preclinical studies have demonstrated the analgesic efficacy of small molecule AT_2_ receptor antagonists in rodent models of peripheral neuropathic pain conditions (Smith et al., 2013a,b, 2014; Muralidharan et al., 2014; Sheahan et al., 2017). These promising preclinical findings have been subsequently translated into a successful Phase 2a proof-of-concept clinical trial outcome (Rice et al., 2014). Specifically, twice-daily oral administration of EMA401, a peripherally restricted AT_2_ receptor antagonist, evoked significant analgesia in a randomized, double-blind, placebo-controlled clinical trial involving 183 patients with post herpetic neuralgia, a type of peripheral neuropathic pain that is difficult to treat (Rice et al., 2014).

Previous work from our laboratory (Smith et al., 2013b) has shown increased expression levels of angiotensin II (Ang II), but not the AT_2_ receptor, in the ipsilateral lumbar (L4–L6) dorsal root ganglia (DRGs) of rats with a chronic constriction injury (CCI) of the sciatic nerve, a widely used rodent model of peripheral neuropathic pain. Importantly, this augmented Ang II/AT_2_ receptor signaling in turn resulted in a marked increase in the expression of phosphorylated-p38 (P-p38) mitogen-activated protein kinase (MAPK) and phosphorylated -p44/p42 (P-p44/p42) MAPK (Smith et al., 2013b). These are key enzymes involved in the phosphorylation of multiple targets implicated in the pathogenesis of peripheral neuropathic pain (Smith et al., 2016). Targets include voltage-gated sodium channels (Na_v_1.7 (Stamboulian et al., 2010) and Na_v_1.8 (Hudmon et al., 2008)), neuronal N-type voltage-gated calcium channels (Ca_v_2.2 (Martin et al., 2006)) and the transient receptor potential cation channel subfamily V member 1 (TRPV1) (Zhuang et al., 2004).

Of particular interest, expression of Ang II and the AT_2_ receptor in the ipsilateral lumbar DRGs of CCI-rats is co-localized with substance P, a marker of small/medium-diameter sensory neurons, and NF200, a marker for medium/large-diameter sensory neurons (Smith et al., 2013b). A role for augmented Ang II signaling via the AT_2_ receptor in sensory neuron hyperexcitability is further supported by the fact that Ang II increased capsaicin-induced excitability of cultured adult rat and human DRG sensory neurons (Anand et al., 2013, 2015). Importantly, this effect was abolished by EMA401 and/or a selective antagonist for Tropomyosin receptor kinase A (TrkA), the high-affinity receptor for NGF (Anand et al., 2015). The latter findings further indicate possible cross-talk between the Ang II/AT_2_ receptor- and the NGF/TrkA-signaling pathways.

However, questions remain on whether the expression of Ang II and the AT_2_ receptor in the ipsilateral lumbar DRGs of CCI-rats is restricted only to neurons or whether they are also expressed by non-neuronal cells, such as CD3^+^ T cells and satellite glial cells (SGCs) that have been implicated by others in the pathophysiology of peripheral neuropathic pain (Hu et al., 2007; Austin et al., 2012; Schmid et al., 2013; Liu et al., 2016). Therefore, the present study was designed to assess the extent to which Ang II is expressed by non-neuronal cells, including CD3^+^ T-cells and SGCs, in the ipsilateral lumbar DRGs of CCI rats and whether or not these putative markers of neuropathic pain are attenuated at the time of peak effect (∼1 h post-dosing) of a single bolus dose (10 mg kg^-1^) of EMA300. Furthermore, our study was also designed to assess the potential for EMA300 to modulate the NGF/TrkA signaling pathway in CCI-rats.

Notably, our present findings show, for the first time, that augmented expression of Ang II in the ipsilateral lumbar DRGs of CCI-rats appears to be underpinned at least in part, by infiltration of Ang II/AT_2_ receptor-expressing CD3^+^ T-cells in addition to the presence of Ang II in SGCs and subsets of neurons. Additionally, in CCI-rats with fully developed mechanical allodynia in the ipsilateral hindpaws, augmented Ang II expression was associated with altered expression levels of both pro-NGF and mature-NGF in the ipsilateral lumbar DRGs. Interestingly, at the time of peak pain relief evoked in CCI-rats by a single intraperitoneal (i.p.) bolus dose of EMA300 at 10 mg kg^-1^, augmented infiltration of Ang II/AT_2_ receptor expressing CD3^+^ T-cells and the altered levels of the mature NGF isoform were reversed in the ipsilateral lumbar DRGs, suggesting that these changes may have a key role in the evoked pain relief.

## Materials and Methods

### Animals

Male Sprague–Dawley rats (220–250 g) were used for all experiments to avoid any possible impact of the estrous cycle on the outcomes of the present study. Animals were obtained from The University of Queensland Biological Resources (UQBR) and were housed in groups of 3–4 per cage in individually ventilated cages in a temperature-controlled facility (22–23°C) with a 12 h/12 h light/dark cycle. Animals were given access to rodent chow and water *ad libitum* except during behavioral observations. All experimental procedures were approved by the ‘Animal Ethics Committee, The University of Queensland’ and performed with adherence to the ‘Australian Code of Practice for the Care and Use of Animals for Scientific Purposes’ (National Health and Medical Research Council [NHMRC], 2013). After arrival in our facility, rats were acclimatized for approximately one week prior to experimentation. Rats (*n* = 35) were randomly assigned to one of the following groups: sham, CCI-vehicle or CCI-EMA300 groups (*n* = 8–15 rats per group).

### Induction of CCI Model

In rats deeply anaesthetized with 3% isoflurane delivered in oxygen, a unilateral CCI was induced by tying four loose ligatures (4–0 silk sutures; Dysilk Black Braided Siliconized Silk, Dynek, Port Adelaide, SA, Australia) at least ∼1 mm apart around one sciatic nerve (Bennett and Xie, 1988; Bennett et al., 2001). In sham-animals, the sciatic nerve was exposed but not ligated. Each rat received subcutaneous benzylpenicillin (60 mg) as antibiotic prophylaxis. The day of surgery was regarded as day 0.

### Assessment of Mechanical Allodynia in the Bilateral Hindpaws

Calibrated Semmes–Weinstein von Frey filaments (Stoelting Co., Wood Vale, IL, United States) were used to determine paw withdrawal thresholds (PWTs) in the ipsilateral (operated side) and contralateral (non-operated side) hindpaws of all rats. Animals were acclimatized for at least 20 min in wire mesh metabolic cages (20 cm × 20 cm × 17 cm) prior to assessment of bilateral PWTs. The von Frey filaments were applied to the plantar surface of the hindpaws to deliver forces in the range 2–20 g at intervals of 2 g using the up-down protocol (Chaplan et al., 1994; Smith et al., 2013a). Animals were randomized prior to behavioral testing by an independent person to ensure that behavioral assessments were conducted in a blinded manner. Temporal development of mechanical allodynia in the ipsilateral hindpaw was assessed once weekly over the 14–17 day study period. The PWTs of the contralateral hindpaws were also assessed during each testing session.

### Administration of EMA300 (Selective AT_2_ Receptor Antagonist) and Vehicle

Between days 14–17 post-surgery, CCI-rats with fully developed ipsilateral hindpaw hypersensitivity (PWTs ≤ 6g) were randomized to receive a single intraperitoneal (i.p.) bolus dose of the sodium salt of EMA300 (also known as PD121981) at 10 mg kg^-1^ (*n* = 15) or vehicle (water for injection; *n* = 8). The bilateral hindpaw PWTs were assessed pre-dose and at ∼1 h post-dose, the time of the EMA300 peak-effect (Smith et al., 2013a,b), following administration of a bolus dose of either EMA300 or vehicle. Sham (control) rats received no treatment, and their PWTs were assessed at similar times to that of the CCI-rats for comparison. All dosing and behavioral assessments were performed by independent personnel in a blinded manner. Specifically, administration of the test-item (EMA300) or vehicle to rats was done by one person whereas the behavioral testing was done by another independent person.

### Tissue Collection and Preparation for *ex Vivo* Analyses

Following assessment of the bilateral hindpaw PWTs at ∼1 h post-EMA300 or vehicle administration, rats were euthanized with an overdose of pentobarbitone (Lethabarb^®^, Virbac, Milperra, NSW, Australia) that was administered i.p. at a dose of ∼300 mg kg^-1^ (i.e., 1 mL kg^-1^ of the stock solution prepared as 325 mg mL^-1^) per rat (Smith et al., 2013b). A group of age-matched sham-control rats (*n* = 12) was also euthanized in a similar manner. Following laminectomy, the ipsilateral lumbar (L4–L6) DRGs were removed from all rats. These tissues were then processed for *ex vivo* investigations using immunohistochemistry (IHC) as well as western blotting (WB). For IHC, rats were perfused with 4% paraformaldehyde (Sigma–Aldrich, Sydney, NSW, Australia) in ice-cold 1x PBS (pH 7.4; ∼150–200 mL per rat) prior to extraction of the lumbar DRGs. Serial cross-sections (8–10 μm thick) were obtained from DRGs embedded in Optimal Cutting Temperature (O.C.T) compound (ProSciTech, QLD, Australia). Superfrost Plus^®^ slides (Thermo Fisher Scientific, Scoresby, VIC, Australia) were used to collect the cryosections. The slides were stored at -20°C until further processing for IHC. For WB, the lumbar DRG tissues were collected without perfusion and were snap-frozen using a dry-ice/ethanol slurry followed by immediate transfer to a -80°C freezer until further experimentation.

### Immunohistochemical Staining

The lumbar DRG sections from all treatment groups were air-dried prior to immunostaining followed by washing with PBS containing 0.05% Tween 20 (Sigma-Aldrich, Sydney, NSW, Australia) (referred herein as PBST) for 2 × 10 min. Subsequently, the sections were incubated for 1–2 h in blocking buffer containing 10% normal goat serum (NGS) (Invitrogen, Mulgrave, VIC, Australia) or 5% Bovine Serum Albumin (BSA) (Sigma–Aldrich) as appropriate for each IHC antibody (Muralidharan et al., 2014; Khan et al., 2015). Later, the sections were incubated with appropriate primary antibodies for at least 15–20 h at ∼4–8°C. Subsequently, the sections were incubated with the corresponding secondary antibodies for ∼1 h at room temperature. The sections were then stained with 4′,6-diamidino-2-phenylindole dihydrochloride (DAPI, Invitrogen) to stain neuronal nuclei followed by mounting with ProLong^®^ Gold anti-fade reagent (Invitrogen). Sections were washed with PBST for at least 2 × 10 min after all antibody incubation steps. The control sections were stained with omission of primary antibodies. To identify the cellular co-localization of Ang II and the AT_2_ receptor in ipsilateral lumbar DRG sections, we used simultaneous immunostaining of these markers with NeuN, CD3, and glial fibrillary acidic protein (GFAP) that are specific markers for neurons, T-cells and SGCs, respectively. The antibodies against the AT_2_ receptor and GFAP were used separately on adjacent sections to avoid cross-reactivity as both antibodies were raised in the rabbit. The images were snapped in the exact location in the region of interest and were overlaid to observe the co-localization. Additionally, we performed co-localization immunostaining for Ang II with the AT_2_ receptor and the AT_2_ receptor with NGF. Prior to the main study, pilot experiments were conducted to optimize the dilutions of each antibody and determine the optimal incubation time, blocking buffer, washing steps and DAPI staining.

The primary antibodies used include, polyclonal guinea pig anti-Angiotensin II (1:200, Peninsula laboratory, CA, United States); polyclonal rabbit anti-AT_2_ receptor (1:500, ab19134, Abcam, Cambridge, MA, United States), monoclonal rabbit anti-AT_2_ receptor (1:500, ab92445, Abcam), monoclonal rat anti-CD3 (1:100, Serotec, Kidlington, United Kingdom); monoclonal rabbit anti-CD3 (1:100, Abcam); monoclonal mouse or rabbit anti-pp38 MAPK and anti-pp44/pp42 MAPK (Cell Signaling Technology, Inc., Danvers, MA, United States); monoclonal rabbit anti-GFAP (1:500, Cell Signaling); rabbit polyclonal anti-NGF (1:500, H-20, Santa Cruz Biotechnology, Santa Cruz, CA, United States); polyclonal sheep anti-NGF (1:500, Abcam); monoclonal rat anti-TrkA (1:50, R&D Systems, Minneapolis, MN, United States) and mouse monoclonal anti-NeuN Alexa Fluor^®^488 conjugated, clone A60 (1:100, Millipore, Temecula, CA, United States). For most of the IHC markers, at least two different antibodies were used as described above to validate the specificity of the immunostaining. Secondary antibodies used include, Alexa Flour 633 goat anti-guinea pig (1:500, Invitrogen); Alexa Flour 546 goat anti-guinea pig (1:800, Invitrogen); Alexa Flour 555 goat anti-sheep (1:800, Invitrogen); Cy3-goat anti-rat (1:800, Jackson ImmunoResearch, Westgrove, PA, United States); Cy3-goat anti-rabbit (1:800, Jackson); Alexa Flour 488 goat anti-rat (1:500, Invitrogen) or Alexa Flour 488 goat anti-rabbit (1:500, Invitrogen). All antibodies were diluted in PBST containing 2% NGS or BSA blocking buffer as appropriate. An Axioskop 40 microscope (Carl Zeiss, Göttingen, Germany) attached to an Axiocam MRm camera (Carl Zeiss) was used for imaging sections at a fixed exposure time that was optimized for each stain in preliminary experiments (Khan et al., 2015).

### Western Blot Analysis

The ipsilateral lumbar DRGs (L4–L6) from each rat in the respective experimental cohort were pooled and homogenized on ice for protein extraction using radio-immunoprecipitation (RIPA) buffer. The buffer was prepared by dissolving 10 mM Tris; 100 mM NaCl, 1%; 1 mM ethylenediaminetetraacetic acid (EDTA); 1mM ethylene glycol tetraacetic acid (EGTA); 1 mM sodium fluoride (NaF); 20 mM tetrasodium pyrophosphate (Na_4_P_2_O_7_); sodium orthovanadate (Na_3_VO_4_); 0.1% sodium dodecyl sulphate (SDS), and 0.5% sodium deoxycholate in MilliQ water followed by addition of 1% Triton X-100 and 10% Glycerol; pH 7.4). The buffer was also supplemented with 1% of each Halt^TM^ Phosphatase Inhibitor Cocktail (Thermo Fisher Scientific, Scoresby, VIC, Australia) and protease Inhibitor Cocktail (Sigma–Aldrich). The protein concentrations were determined with the Bradford method (protein assay kit, Bio-Rad Laboratories, Milan, Italy). From each extract, 30 μg of total protein was loaded onto the 4–20% Mini-PROTEAN^®^ TGX Stain-Free^TM^ precast polyacrylamide gels (Bio-rad, Gladesville, NSW, Australia). Each gel was then transferred to a 0.45 μm Immobilon-FL PVDF membrane (Millipore) and incubated in Odyssey^TM^ blocking buffer (Li-cor, Lincoln, NE, United States) for at least 1 h. For phospho-specific antibodies, the blocking buffer comprised 5% BSA/0.1% Tween-20. The membranes were incubated overnight at ∼4°C with specific antibodies against the AT_2_ receptor (1:500, ab92445, Abcam), GFAP (1:300, Cell signaling), NGF (1:500, H-20), TrkA (1:500, R&D Systems), phospho-p38 MAPK (pp38 MAPK), phospho-p44/pp42 MAPK, p38 MAPK (total p38 MAPK), and p44/p42 MAPK (total extracellular signal-related kinases (ERK)) (each 1:1000). The sources of primary antibodies are the same as listed in section “Immunohistochemical Staining”. Monoclonal mouse anti-beta actin (1:10,000, Abcam) (1:8000, Novus, Littleton, CO, United States) was used as the loading control. Following washing with PBST (3 × 5 min), the membranes were incubated in the appropriate IRDye^®^ secondary antibodies (Li-cor) (1:8000) for ∼1 h at room temperature. The Odyssey^®^ CLx imaging system (Li-cor) was used to visualize the membranes.

### Cell Culture, Transfection, and Tunicamycin Treatment

Human embryonic kidney (HEK) cells obtained from the American Type Culture Collection (ATCC, VA United States) were transfected with rat AT_1_ receptor complementary DNA (cDNA) (HEK-AT_1_) alone or rat AT_2_ receptor cDNA (HEK-AT_2_) alone using Lipofectamine^®^ 2000 as per the manufacturer’s protocol. Dulbecco’s Modified Eagle’s Medium (DMEM) (Invitrogen) supplemented with 10% Fetal bovine serum (FBS) (Invitrogen) was used for culturing these cell lines. For transfected cell-lines, G418 (Geneticin) (500 mg mL^-1^) was added to the growth medium as a selective agent for generating cell lines stably expressing either the AT_2_ receptor or the AT_1_ receptor. These stable cell lines were maintained at 37°C in a 5% humidified CO_2_ incubator. To assess if the disparity between the molecular mass of the AT_2_ receptor (kDa) from the DRG tissues and that from HEK-AT_2_ cells were potentially related to different degrees of N-glycosylation, the cells were incubated with tunicamycin (TNM) (Sigma–Aldrich), an inhibitor of N-glycosylation. Specifically, the HEK-AT_2_ cells were incubated with TNM at 1 or 3 μg mL^-1^ for 48 h, conditions previously shown by others to not affect cell viability (Servant et al., 1996). The HEK-AT_2_- as well as HEK-AT_1_ -cells not treated with TNM served as positive and negative control groups, respectively. The cells were lysed with RIPA buffer and processed for WB analysis using the monoclonal rabbit anti-AT_2_ receptor antibody (ab92445, Abcam) as described in preceding section.

### Immunocytochemistry

In order to confirm the specificity of the monoclonal rabbit anti-AT_2_ receptor (Abcam; ab92445), HEK-cells stably expressing either the AT_2_ or the AT_1_ receptor were cultured using the identical procedure described in preceding section. Briefly, the cells were grown to 50–70% confluency on sterilized coverslips placed in 6-well plates. Following harvest, the cells were fixed with ice-cold 100% methanol for 5 min at room temperature. Subsequently, the cells were washed with PBST (3 × 5 min) and blocked with 10% NGS in PBST for 30 minutes at room temperature. The cells were then incubated overnight with each monoclonal rabbit anti-AT_2_ receptor antibody (ab92445 and ab19134, Abcam). For visualization, Alexa Flour 546 goat anti-rabbit (1:800, Invitrogen) was used and it was incubated with cells for at least 1 h. The cells were then incubated with DAPI and were mounted using ProLong^®^ Gold anti-fade reagent prior to imaging using similar washing steps as described in section “Immunohistochemical Staining”. The cells incubated with only secondary antibody served as an internal experimental control. Un-transfected HEK-cells were used as an additional negative control. The HEK-AT_2_ cells stained with anti-AT_2_ receptor antibody (Abcam, ab19134), the specificity of which was previously validated using adrenal and DRG tissues from AT_2_ receptor knockout mice (Smith et al., 2013b), served as a positive control.

### Data Analyses

Behavioral data (PWT values) are presented as mean (±SEM) of three measurements taken at least 5 min apart individually for the ipsilateral and contralateral hindpaws. For IHC, the Axiovision Rel. v4.8 software (Carl Zeiss, Göttingen, Germany) was used to determine the mean fluorescence intensity. The IHC quantification was performed on at least four lumbar L4–L6 DRG sections (>100 μm apart) obtained from 3 to 4 rats in each treatment group. IHC quantification data is expressed as fold-changes in fluorescence intensity relative to that for the corresponding sections from sham-rats. ImageJ 1.48v (NIH, United States) was used for counting CD3^+^ T-cells with or without Ang II co-expression. For western blot analysis, Image Studio Lite v4.0 software (Li-cor, Lincoln, NE, United States) was used to quantify the band intensities for each treatment group using 3–4 rats per group. Band intensities were normalized relative to beta-actin and the results are shown relative to the corresponding data from sham-control rats.

### Statistical Analyses

To analyze the pain behavioral end-points, we used two-way analysis of variance (ANOVA) with a *post hoc* Bonferroni test for multiple comparisons. The one-way ANOVA followed by Tukey’s multiple comparison test was used to analyze the between-group differences for IHC and WB. GraphPad Prism^TM^ v7 (GraphPad Software, La Jolla, United States) was used for statistical analyses. The statistical significance criterion was *P* ≤ 0.05.

## Results

### Temporal Development of Mechanical Allodynia in the Ipsilateral Hindpaws of CCI-Rats and Its Alleviation by EMA300

**FIGURE 1:**
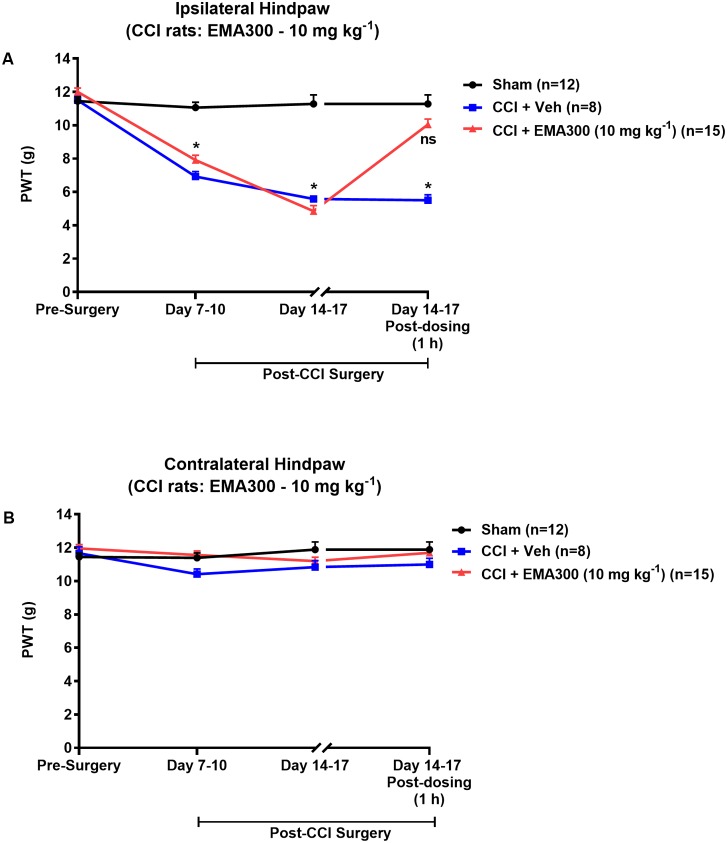
Hypersensitivity in the ipsilateral hindpaws of chronic constriction injury (CCI)-rats was alleviated by a bolus dose of EMA300. **(A)** Mechanical allodynia (PWTs ≤ 6 g) was developed in the ipsilateral hindpaws of CCI-rats by day 7–10 post-surgery and it was maintained until at least day 14–17 post-surgery. In CCI-rats treated with a single i.p. bolus dose of EMA300 (10 mg kg^-1^) but not vehicle, there was significant relief (*F*_(2,3, 6/96)_ = 51.66, 115.7, 43.65; *P ≤* 0.05) of ipsilateral hindpaw hypersensitivity so that the PWTs matched those of sham-rats at the time of peak analgesia (∼1 h post dosing) evoked by EMA300. **(B)** In the contralateral hindpaws of CCI-rats, hindpaw hypersensitivity did not develop and EMA300, like vehicle was without effect (*F*_(2,3,6/96)_ = 2.11, 2.66, 1.88; *P >* 0.05). All PWT values in **(A,B)** are the mean ± SEM from 8 to 15 rats per group. Asterisks indicate statistically significant differences (*P ≤* 0.05) compared to the corresponding values for sham rats using a two-way repeated measures analysis of variance (ANOVA) with a *post hoc* Bonferroni test for multiple comparisons.

Mechanical allodynia (PWTs ≤ 6 g) in the ipsilateral hindpaws of CCI-rats was fully developed by day 14–17 post-surgery (**Figure 1A**). By contrast, mechanical allodynia did not develop in the contralateral hindpaws of CCI-rats or in the bilateral hindpaws of the sham-group rats (**Figures 1A,B**). Following administration of a single i.p. bolus dose of EMA300 (10 mg kg^-1^) to CCI-rats with fully developed ipsilateral hindpaw hypersensitivity, there was significant [F_(2,3,6/96)_ = 51.66, 115.7, 43.65; *P ≤* 0.05] relief of mechanical allodynia in the ipsilateral hindpaws at ∼1 h post-dosing (time of peak-effect), whereas vehicle (saline)-treatment was inactive (**Figure 1A**). These findings are in agreement with previous work from our laboratory (Smith et al., 2013a,b). Importantly, single i.p. bolus doses of EMA300 (10 mg kg^-1^) did not result in any discernible central nervous system (CNS) side-effects, for example, sedation, or neuro-excitatory behaviors, during all assessments.

### Co-localization of Ang II and/or the AT_2_ Receptor with CD3^+^ T-Cells and SGCs in Addition to Sensory Neuronal Markers

**FIGURE 2:**
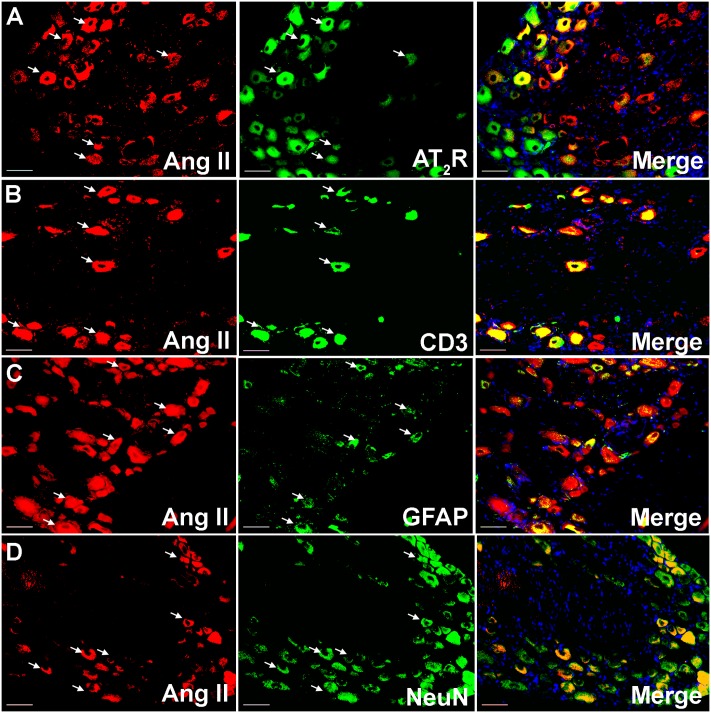
Double immunostaining to assess the potential cellular source of Ang II in the ipsilateral lumbar DRGs (L4–L6) of vehicle treated CCI-rats. Ang II was co-localized with **(A)** the AT_2_ receptor **(B)** CD3^+^ T-cells **(C)**, GFAP and **(D)** subsets of neurons (NeuN). Merged images also contain DAPI (blue color) stained nuclei. Scale bars = 50 μm.

**FIGURE 3:**
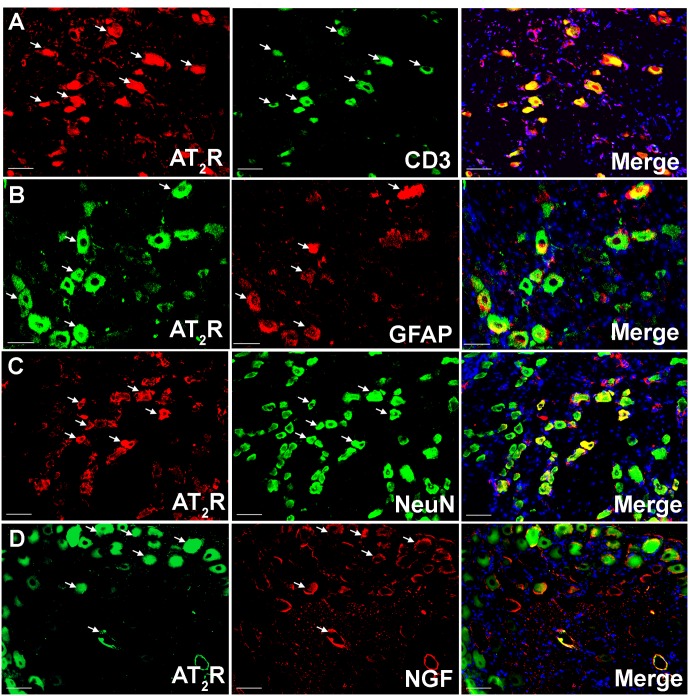
Double immunostaining to investigate the cellular location of the AT_2_ receptor in the ipsilateral lumbar DRGs (L4–L6) of vehicle treated CCI-rats. The AT_2_ receptor was co-localized with **(A)** CD3^+^ T-cells **(B)** GFAP **(C)** subsets of neurons (NeuN), and **(D)** NGF expressing neurons suggesting cross-talk of augmented Ang II-AT_2_ receptor signaling with the augmented NGF-TrkA signaling pathway. Merged images also contain DAPI (blue color) stained nuclei. Scale bars = 50 μm.

Using IHC, our findings show that Ang II and the AT_2_ receptor are co-localized with CD3^+^ T-cells and SGCs (GFAP) in the ipsilateral lumbar DRG sections from CCI-rats exhibiting neuropathic pain behavior (**Figures 2**, **3**). Additionally, in agreement with our previous observations (Smith et al., 2013b; Muralidharan et al., 2014), Ang II and/or the AT_2_ receptor were also expressed by subsets of sensory neurons marked by neuronal nuclei protein (NeuN) in addition to NGF in the ipsilateral lumbar DRGs of CCI-rats (**Figures 2**, **3**).

### Increased Number of Ang II-Expressing CD3^+^ T-Cells in the Ipsilateral Lumbar DRGs of CCI-Rats Is Attenuated by EMA300

**FIGURE 4:**
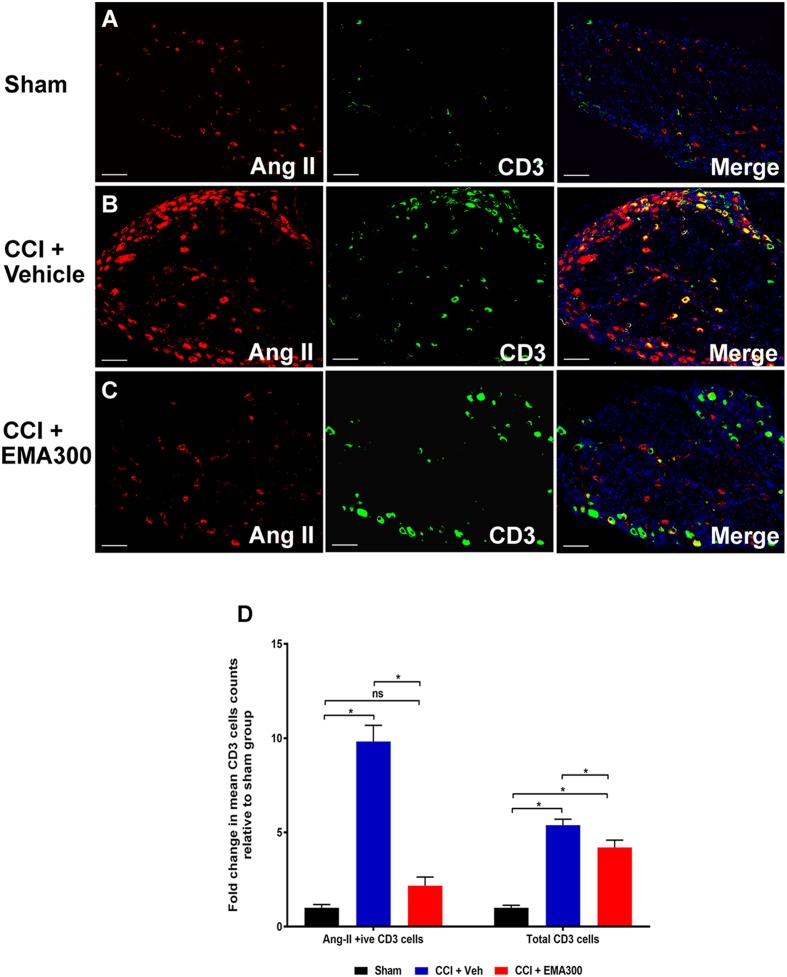
Immunofluorescence staining and quantification of Ang II expressing (Ang II+ive) CD3^+^ T-cells in sections of the ipsilateral lumbar DRGs (L4–L6) from CCI-rats administered a bolus dose of EMA300 (10 mg kg^-1^) or vehicle relative to the corresponding sections from sham-rats **(A–C)** Representative images from each group show co-localization of Ang II with CD3^+^ T-cells **(D)** The number of Ang II+ive CD3^+^ T-cells as well as total CD3^+^ T-cells were increased by ∼9.8-fold (*F*_(2,33)_ = 64.1; *P ≤* 0.05) and ∼5.3 (*F*_(2,33)_ = 53.37; *P ≤* 0.05), respectively, in vehicle-treated CCI-rats that were significantly reduced by EMA300. Following treatment with EMA300, the number of Ang II+ive CD3^+^ T-cells but not total CD3^+^ T-cells matched the corresponding data from sham-rats (One-way ANOVA followed by Tukey’s multiple comparison test). Scale bars represent 100 μm. CCI, Chronic constriction injury, Veh, vehicle.

Between days 14–17 post-surgery, there was a significant increase in the number of Ang II-expressing CD3^+^ T-cells (∼9.8-fold; *F*_(2,33)_ = 64.1; *P ≤* 0.05) as well as the total number of CD3^+^ T-cells (∼5.3-fold; *F*_(2,33)_ = 53.37; *P ≤* 0.05) in the ipsilateral lumbar DRG sections of vehicle-treated CCI-rats relative to sham-group rats (**Figure 4**). Interestingly, administration of a single i.p. bolus dose of EMA300 (10 mg kg^-1^) to CCI-rats significantly reduced the otherwise increased number of CD3^+^ T-cells expressing Ang II (*F*_(2,33)_ = 64.1; *P >* 0.05) and the total number of CD3^+^ T-cells (*F*_(2,33)_ = 53.37; *P ≤* 0.05) at its time of peak analgesic effect (∼1 h post-dosing). In particular, the number of Ang II-expressing CD3^+^ T-cells in the ipsilateral lumbar DRGs of CCI-rats treated with EMA300 (10 mg kg^-1^) was reduced to match the number in the corresponding sections from the sham-group of rats (**Figure 4**).

### Specificity of the AT_2_ Receptor Antibody (ab92445 Compared with ab19134) Using WB and ICC Techniques

**FIGURE 5:**
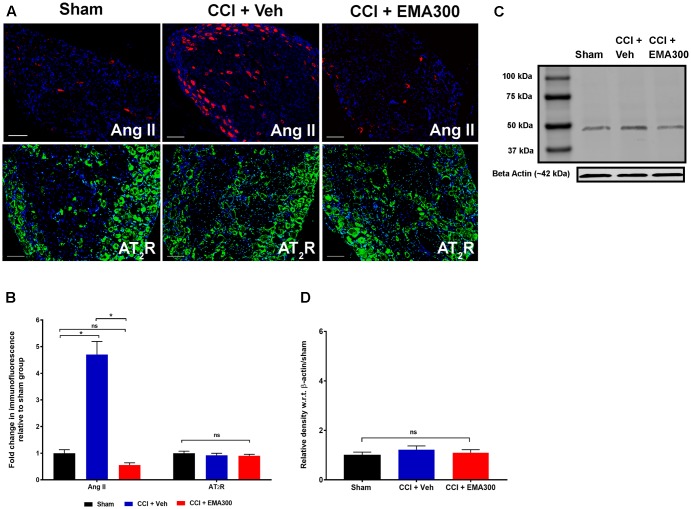
Immunofluorescence and/or western blot analysis of Ang II and the AT_2_ receptor in sections of the ipsilateral lumbar DRGs (L4–L6) from CCI-rats administered a single bolus dose of EMA300 (10 mg kg^-1^) or vehicle relative to the corresponding sections from sham-rats. **(A,B)** Ang II was increased by ∼4.7 fold (*F*_(2,33)_ = 58.65; *P ≤* 0.05) in the ipsilateral lumbar DRGs from vehicle-treated CCI-rats and this was effectively inhibited by EMA300 treatment. Expression levels of the AT_2_ receptor were not significantly altered among all treatment groups using **(A,B)** immunofluorescence (*F*_(2,44)_ = 0.6060; *P >* 0.05) and **(C,D)** western blot analyses (*F*_(2,6)_ = 0.69; *P >* 0.05). (One-way ANOVA followed by Tukey’s multiple comparison test). (One-way ANOVA followed by Tukey’s multiple comparison test). Scale bars represent 100 μm. CCI, Chronic constriction injury, kDa, kiloDaltons; Veh, vehicle.

Herein, we found that the polyclonal rabbit anti-AT_2_ receptor antibody (ab19134), previously validated for IHC using adrenal gland and DRG sections from AT_2_ receptor knockout mice (Smith et al., 2013b), is not compatible for use with western blotting (data not shown). Therefore, we used a monoclonal rabbit anti-AT_2_ receptor antibody (ab92445) for western blots and validated its specificity for the AT_2_ receptor using western blotting and immunocytochemistry (ICC) (**Supplementary Figure S1**). Our results demonstrate that the monoclonal rabbit anti-AT_2_ receptor antibody (ab92445) bound specifically to HEK-cells stably expressing the AT_2_ receptor, but not to un-transfected HEK-cells or to HEK-cells stably expressing the AT_1_ receptor (**Supplementary Figure S1**). In ICC, the polyclonal rabbit anti-AT_2_ receptor antibody (ab19134) was used as a positive control antibody to corroborate the results obtained using the monoclonal rabbit anti-AT_2_ receptor antibody (ab92445). During the western blot analysis of the HEK-AT_2_ cells, the expression of the AT_2_ receptor was observed at ∼90 kDa (**Supplementary Figure S1**). In contrast, the AT_2_ receptor was expressed at ∼50 kDa for the ipsilateral lumbar DRGs samples from CCI- and sham-rats (**Figure 5C**). A possible explanation of this paradoxical observation may be due to differential levels of glycosylation, which are frequently associated with the AT_2_ receptor (Servant et al., 1994, 1996). In support of this notion, following de-glycosylation with tunicamycin (TNM) at 1 and 3 μg mL^-1^ for 48 h, the band for the AT_2_ receptor shifted from 90 to ∼50 kDa, to match that of the AT_2_ receptor in DRG tissue samples (**Supplementary Figure S1**).

### Effect of EMA300 on the Expression Levels of Ang II and the AT_2_ Receptor in the Ipsilateral Lumbar DRGs of CCI-Rats

In agreement with previous work from our laboratory (Smith et al., 2013b), there was a ∼4.7-fold increase in the Ang II IF (*F*_(2,33)_ = 58.65; *P* ≤ 0.05) in the ipsilateral lumbar DRGs of CCI-rats with fully developed ipsilateral hindpaw hypersensitivity *c.f.* the corresponding sections from sham-group rats (**Figures 5A,B**). At the time of peak analgesia evoked by a single i.p. bolus dose of EMA300 at 10 mg kg^-1^, the elevated expression levels of Ang II IF in the CCI-rats were markedly decreased (*F*_(2,33)_ = 58.65; *P* ≤ 0.05) to match the corresponding levels in sham-rats (**Figures 5A,B**).

There was no significant difference (*P >* 0.05) in the expression levels of the AT_2_ receptor in sections of ipsilateral lumbar DRGs from CCI-rats administered a single i.p. bolus dose of either EMA300 or vehicle using IHC (*F*_(2,44)_ = 0.6060, **Figures 5A,B**) and western blot (*F*_(2,6)_ = 0.6946, **Figures 5C,D**).

### Disruption of NGF Isoforms in Ipsilateral Lumbar DRGs of CCI Rats: Amelioration of Mature-NGF Isoform by EMA300

**FIGURE 6:**
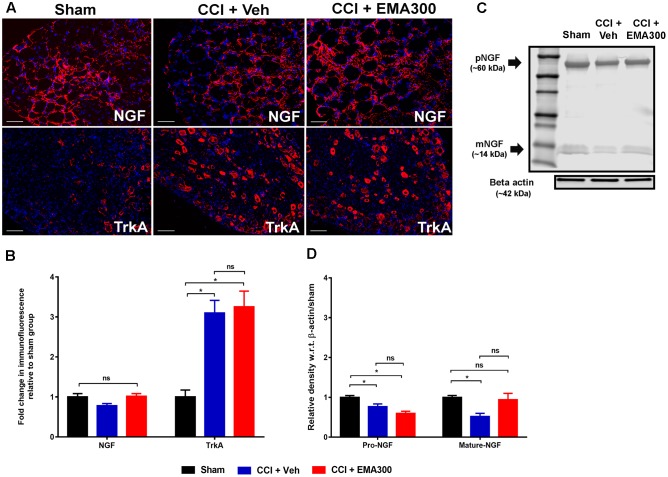
Immunofluorescence and/or western blot analyses of NGF and TrkA in sections of the ipsilateral lumbar DRGs (L4–L6) from CCI-rats administered a single bolus dose of EMA300 (10 mg kg^-1^) or vehicle relative to the corresponding sections from sham-rats. **(A,B)** During IHC, the expression level of total NGF was not significantly altered in CCI-rats among all treatment groups (*F*_(2,31)_ = 3.69; *P >* 0.05) relative to sham-rats. However, the expression of TrkA was elevated by ∼3-fold (*F*_(2,28)_ = 14.37; *P ≤* 0.05) in vehicle-treated CCI rats that was not changed by EMA300. **(C,D)** The western blot analyses of NGF revealed that in the ipsilateral lumbar DRGs of CCI-rats, expression of the pro- and mature-NGF isoforms were significantly reduced (*F*_(2,9)_ = 11.95; *P ≤* 0.05) and that expression of mature-NGF was rescued at the time of peak effect of a single i.p. bolus dose of EMA300 (10 mg kg^-1^) to match the corresponding mature NGF expression level in sham-rats. (One-way ANOVA followed by Tukey’s multiple comparison test). Scale bars represent 50 μm. CCI, Chronic constriction injury, kDa, kiloDaltons; Veh, vehicle.

Herein, total NGF IF did not differ significantly (*F*_(2,33)_ = 3.695; *P >* 0.05) in the ipsilateral lumbar DRG sections from CCI- and sham-rats (**Figures 6A,B**). However, there were significant differences for the individual NGF isoforms when assessed by WB such that these isoforms were significantly reduced (pro-NGF, *F*_(2,9)_ = 13.12; mature-NGF, *F*_(2,9)_ = 5.944; *P ≤* 0.05) in vehicle-treated CCI-rats compared with sham-rats (**Figures 6C,D**). Notably, we show for the first time that a single i.p. bolus dose of EMA300 completely restored the otherwise significantly reduced (*F*_(2,9)_ = 11.95; *P ≤* 0.05) mature-NGF expression levels in the ipsilateral lumbar DRGs of CCI rats at the time point of its peak analgesia (**Figures 6C,D**). Additionally, there was no significant change in pro-NGF expression in the ipsilateral lumbar DRGs of CCI-rats treated with EMA300 when compared with the vehicle treated CCI group (**Figures 6C,D**).

### EMA300 Did Not Inhibit the Upregulated Levels of TrkA in the Ipsilateral Lumbar DRGs of CCI Rats

In the somatosensory DRG neurons from animal models, the high affinity NGF receptor, TrkA, is implicated in the pathophysiology of neuropathic pain (Grelik et al., 2005; Ugolini et al., 2007). In agreement with previous reports by others (Fang et al., 2005), we also observed that there was an ∼3-fold increase (*F*_(2,28)_ = 14.37; *P ≤* 0.05) in TrkA expression in the ipsilateral lumbar DRG sections of CCI-rats with fully developed hindpaw hypersensitivity *c.f.* the corresponding sections from sham-rats (**Figures 6A,B**). However, the elevated TrkA expression levels were not altered by a single bolus dose of EMA300 (**Figures 6A,B**). These findings suggest that the acute anti-allodynic effects of EMA300 in CCI rats is not transduced by a change in TrkA expression.

### Activation of GFAP-Expressing SGCs in the Ipsilateral Lumbar DRGs of CCI Rats Were Not Significantly Altered by EMA300

**FIGURE 7:**
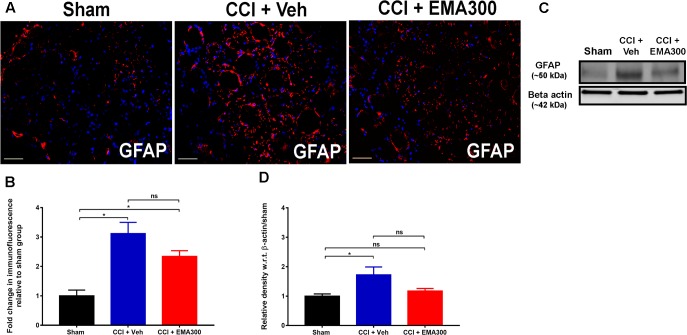
Immunofluorescence and western blot analyses of GFAP expression in sections of the ipsilateral lumbar DRGs (L4–L6) from CCI-rats administered a single bolus dose of EMA300 (10 mg kg^-1^) or vehicle relative to the corresponding sections from sham-rats. **(A,B)** GFAP IF was augmented by ∼3.1-fold (*F*_(2,33)_ = 15.23; *P ≤* 0.05) in the ipsilateral lumbar DRGs of CCI-rats treated with vehicle; however, it was not significantly altered during the acute pain relief produced by single bolus dose of EMA300 (10 mg kg^-1^) relative to sham-rats. **(C,D)** WB analyses also confirmed that GFAP expression was elevated by ∼1.7-fold (*F*_(2,9)_ = 5.398; *P ≤* 0.05) in CCI rats administered vehicle, however, it was not significantly (*P >* 0.05) altered in CCI-rats following treatment with EMA300 relative to sham-rats (One-way ANOVA followed by Tukey’s multiple comparison test). Scale bars represent 50 μm. CCI, Chronic constriction injury, kDa, kiloDaltons; Veh, vehicle.

Consistent with previous work by others (Hu et al., 2007; Liu et al., 2016), we found a significant increase in GFAP IF (IHC ∼3.1 fold, *F*_(2,33)_ = 15.23; WB ∼1.7-fold, *F*_(2,9)_ = 5.398; *P ≤* 0.05) in sections of the ipsilateral lumbar DRGs of vehicle-treated CCI rats relative to the corresponding sections from sham-rats (**Figure 7**). However, at the time of peak effect of a single i.p. bolus dose of EMA300 (10 mg kg^-1^), there was insignificant attenuation (*P >* 0.05) of GFAP IF suggesting that the anti-allodynic effect of acutely administered EMA300 in CCI-rats is not mediated by regulation of GFAP in the ipsilateral lumbar DRGs of CCI-rats (**Figure 7**).

### Augmented MAPK-Signaling in Ipsilateral Lumbar DRGs of CCI-Rats and Its Inhibition by EMA300

**FIGURE 8:**
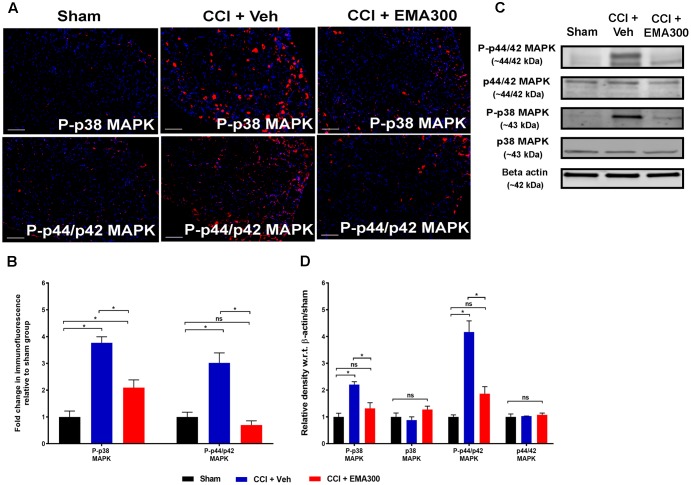
Immunofluorescence and western blot analyses of P-p38 MAPK and P-p44/p42 MAPK in sections of the ipsilateral lumbar DRGs (L4–L6) from CCI-rats administered a bolus dose of EMA300 (10 mg kg^-1^) or vehicle relative to the corresponding sections from sham-rats. **(A,B)** During IHC, expression levels of phospho-p38 (P-p38) MAPK and phospho-p44/p42 (P-p44/p42) MAPK were increased by ∼3.7-fold (*F*_(2,26)_ = 21.01; *P ≤* 0.05), and ∼3-fold (*F*_(2,6)_ = 33.58; *P ≤* 0.05), respectively, compared with sham-rats. These augmented downstream mediators of Ang II-AT_2_ signaling were significantly reduced by EMA300 treatment. **(C,D)** WB analysis also confirmed that the expression of phospho-p38 (P-p38) MAPK and phospho-p44/p42 (P-p44/p42) MAPK were increased by ∼2.2-fold (*F*_(2,6)_ = 21.01; *P ≤* 0.05) and ∼4.1-fold (*F*_(2,6)_ = 33.58; *P ≤* 0.05), respectively. A single bolus dose of EMA300 at 10 mgkg^-1^ significantly reduced expression levels of MAPK (P-p38 and P-p44/p42) compared with the sham-control group. On the other hand, the expression levels of total p38 MAPK (*F*_(2,6)_ = 2.471; *P >* 0.05) or total p44/p42 MAPK (*F*_(2,6)_ = 0.3029; *P >* 0.05) were not changed in the ipsilateral lumbar DRGs of CCI-rats relative to the corresponding data from sham-rats. (One-way ANOVA followed by Tukey’s multiple comparison test). Scale bars represent 100 μm. CCI, Chronic constriction injury, kDa, kiloDaltons; Veh, vehicle.

Consistent with previous work from our laboratory (Smith et al., 2013b), there was a significant increase in P-p38 MAPK (IHC ∼3.7-fold, *F*_(2,6)_ = 21.01; WB ∼2.2-fold, *F*_(2,6)_ = 21.01; *P ≤* 0.05) and P-p44/p42 MAPK (IHC ∼3-fold, *F*_(2,6)_ = 33.58; WB ∼4.1-fold, *F*_(2,6)_ = 33.58; *P ≤* 0.05) in the ipsilateral lumbar DRGs of vehicle-treated CCI rats compared with the corresponding values for sham rats (**Figure 8**). Notably, the augmented levels of P-p38 MAPK and MAPK were reduced in CCI rats following a single bolus i.p. dose of EMA300 to match the respective level in sham-rats (**Figure 8**).

## Discussion

In the present study, we show for the first time that for CCI-rats exhibiting robust mechanical allodynia in the ipsilateral hindpaws there was an ∼9.8-fold increase in the number of Ang II-expressing CD3^+^ T-cells in their ipsilateral lumbar DRGs. This increase was reversed at the time of peak pain relief produced at ∼1 h after administration of a single i.p. bolus dose of EMA300 (10 mg kg^-1^) in these rats. Additionally, we found an ∼5.3-fold increase in the total number of T-cells in the ipsilateral lumbar DRGs and this number was also reduced significantly at the time of peak effect of a single bolus dose of EMA300. Together, these findings extend previous work by others that demonstrated infiltration of peripheral immune cells including T-cells into the lumbar DRGs of rats with a chronic compression injury to one sciatic nerve (Hu et al., 2007; Costigan et al., 2009; Schmid et al., 2013; Austin et al., 2015; Barr et al., 2016).

Our present data show that Ang II and the AT_2_ receptor are not only expressed by sensory neurons in the lumbar DRGs (Smith et al., 2013b; Benitez et al., 2017), but also by non-neuronal cells including SGCs and CD3^+^ T-cells. Therefore, it is reasonable to suggest that EMA300 has possibly reduced the infiltration of Ang II expressing CD3^+^ T-cells in the ipsilateral lumbar DRGs of CCI rats by blocking autocrine and paracrine activation of these immune cells that express Ang II as well as the AT_2_ receptor (Hoch et al., 2009). This notion is reinforced by previous work by others demonstrating that infusion of Ang II at 490 ng kg^-1^min^-1^ to C57BL/6 mice for 2 weeks significantly increased the infiltration of CD3^+^ T-cells into the renal cortex (Boesen et al., 2011). In other work, an Ang II-dependent increase in the influx of CD3^+^ T-cells/monocytes into the rodent kidney (Wolf et al., 1997) was attenuated by an AT_2_ receptor antagonist (Wolf et al., 1997).

Until the recent reports from our laboratory implicating augmented Ang II signaling by the AT_2_ receptor in the pathophysiology of neuropathic pain (Smith et al., 2013a,b, 2014), a role for the AT_2_ receptor remained enigmatic (Rice and Smith, 2015; Smith et al., 2016). Our previous reports revealed for the first time that selective small molecule AT_2_ receptor antagonists, e.g., EMA200, EMA300, EMA400, evoked dose-dependent relief of mechanical allodynia in the ipsilateral hindpaws of CCI rats (Smith et al., 2013a) that was abolished in mice null for the AT_2_ receptor (Smith et al., 2013b). Given that the AT_2_ receptor does not modulate blood pressure *in vivo* (Porrello et al., 2009), and that the clinical candidate, EMA401 (S-enantiomer of EMA400) has >10,000-fold binding selectivity for the AT_2_ receptor over the AT_1_, and it does not cross the blood-brain-barrier (Anand et al., 2013), it has attracted considerable attention as a clinically validated therapeutic target for relief of peripheral neuropathic pain conditions (Finnerup and Baastrup, 2014; Rice et al., 2014; Smith et al., 2016). A role for the AT_2_ receptor in the pathobiology of peripheral neuropathic pain is supported by *in vitro* work by others whereby the AT_2_ receptor modulated neuronal excitability and neurite outgrowth evoked by Ang II in cultured cells of neuronal origin (Li et al., 2005; Guimond and Gallo-Payet, 2012a,b; Namsolleck et al., 2014) as well as cultured adult human DRG neurons (Anand et al., 2013, 2015). Of particular note, the neurite outgrowth in the cultured adult rat DRG neurons was significantly increased in the presence of Ang II or the AT_2_ receptor agonist, EMA1087 (Compound 21), and that was reversed by the AT_2_ receptor antagonist (EMA401), without producing neurotoxicity (Anand et al., 2013, 2015).

Due to specificity concerns on some commercially available anti-AT_2_ receptor antibodies, we validated the specificity of the AT_2_ receptor antibodies used for IHC and western blot analyses herein (Hafko et al., 2013). In our previous work (Smith et al., 2013b), we used cryosections from adrenal gland and lumbar DRGs of wild-type mice and AT_2_ receptor knock-out mice to show the specificity of an Abcam AT_2_ receptor antibody (ab19134, Abcam). However, this AT_2_ receptor antibody (ab19134) was unsuitable for western blot analysis as indicated by its supplier (Abcam) as it is not uncommon for the binding epitopes of a target protein to be masked in one technique but not another (Mahmood and Yang, 2012; Marx, 2013). To address this issue, we used HEK-cells stably transfected with either the AT_1_ receptor or the AT_2_ receptor to assess the specificity of a rabbit monoclonal anti-AT_2_ receptor antibody (ab92445) that specifically bound to the AT_2_ receptor and not the AT_1_ receptor during ICC and WB experiments herein. As the AT_2_ receptor is a glycoprotein that has five potential N- glycosylation sites located at the extracellular N-terminus of the receptor (Servant et al., 1994), its molecular size (kDa) has the potential to vary in the range, ∼50 to ∼90 kDa, in western blot analyses depending upon the extent to which it is N-glycosylated (Servant et al., 1994). In agreement with others, we showed that incubation of HEK-AT_2_ cells with low concentrations of tunicamycin (1 and 3 μg mL^-1^ for 48 h), a well-known inhibitor of N-glycosylation (Servant et al., 1994, 1996), shifted the band for the AT_2_ receptor from ∼90 to ∼50 kDa with the latter mimicking the AT_2_ receptor band in western blots of rat lumbar DRGs.

Strikingly herein, we also found that the expression levels of pro- and mature-isoforms of NGF were significantly reduced in the ipsilateral lumbar DRGs of vehicle-treated CCI-rats compared with sham rats. Intriguingly, the reduced expression of mature-NGF but not pro-NGF was normalized by EMA300 (10 mg kg^-1^) with it being comparable to its corresponding expression levels in sham-rats at the time of peak EMA300 analgesia. These results were confirmed by using two different types of anti-NGF antibodies that were able to differentially identify both isoforms of NGF (Barcelona and Saragovi, 2015). The significantly reduced expression of mature-NGF in the ipsilateral lumbar DRGs of CCI rats may be due to defective maturation of pro-NGF as shown by others in brain tissue of diabetic rats (Khan and Smith, 2015; Soligo et al., 2015). In work by others, there was a significant decrease in NGF expression in the lumbar DRGs of CCI rats during an early stage that was recovered gradually over time (Lee et al., 1998). In contrast, glaborous skin tissues from the ipsilateral hindpaws of CCI rats showed increased expression of pro-NGF in CCI-rats compared with sham animals (Peleshok and Ribeiro-da-Silva, 2012), but the expression levels of mature-NGF were not reported.

In agreement with others (Fang et al., 2005; Grelik et al., 2005), our present findings show an upregulation of the expression of TrkA, the high affinity receptor for NGF in the ipsilateral lumbar DRGs of CCI rats. However, the elevated expression levels of TrkA were not decreased by a single-bolus dose of EMA300. These observations along with the co-localization of the AT_2_ receptor with NGF suggest a potential cross-talk between augmented Ang II/AT_2_ receptor signaling and that of the NGF/TrkA pathway. In support of this notion, work by others has shown that Ang II-induced activation of ERK (p44/p42) phosphorylation was decreased following co-stimulation with NGF when compared with Ang II or NGF alone (Yamada et al., 1996; Stroth et al., 2000). Given that NGF is also expressed by SGCs in addition to that by neuronal cell bodies (Lee et al., 1998), future studies are warranted to investigate possible differential temporal changes in NGF expression patterns in various peripheral neuropathic pain conditions and/or their possible modulation by AT_2_ receptor antagonists.

We also observed herein a significant increase in GFAP expression representing SGC activation in the ipsilateral lumbar DRGs of CCI-rats that was unaltered by the single bolus dose of EMA300 (10 mg kg^-1^). Our observations are aligned with previous work by others showing that systemic administration of a sub-pressor dose of Ang II (150 ng kg^-1^min^-1^) for 7 days potentiated hindpaw hypersensitivity in CCI-rats and that this was accompanied by Ang II mediated SGC activation in the ipsilateral lumbar DRGs of these animals (Pavel et al., 2013).

Mirroring our previous findings in the ipsilateral lumbar DRGs of CCI-rats (Smith et al., 2013b), our present data show that at the time of peak pain relief evoked by a single bolus dose of EMA300 at 10 mg kg^-1^ in CCI-rats, there was a significant reduction in the otherwise augmented Ang II/AT_2_ receptor signaling and its downstream mediators, P-p38 MAPK and P-p44/p42 MAPK. Our findings herein are also aligned with recent work by others showing that incubation of cultured rat DRG neurons with Ang II (10 nM) for 30 min considerably increased immunofluorescence for P-p38 MAPK and P-p44/p42 MAPK expression (Anand et al., 2015). Interestingly, the upregulated expression levels of these MAPKs in cultured rat DRG neurons were significantly reversed by the small molecule AT_2_ receptor antagonist, EMA401 at 100 nM (Anand et al., 2015).

## Conclusion

A single bolus dose of EMA300, a selective small molecule AT_2_ receptor antagonist, alleviated hindpaw hypersensitivity in the ipsilateral hindpaws of CCI-rats. This was accompanied by attenuation of otherwise augmented infiltration of Ang II-expressing CD3^+^ T-cells into the ipsilateral lumbar DRGs of CCI-rats. Concurrently, the acute analgesic effect of EMA300 appeared to be mediated by restoration of the otherwise reduced expression levels of the mature NGF isoform in the ipsilateral lumbar DRGs of CCI-rats to match that in the corresponding DRG sections from sham rats. Furthermore, EMA300 significantly reduced the elevated expression of the downstream signaling cascade of Ang II/AT_2_ receptor particularly P-p38 MAPK and P-p44/p42 MAPK in the ipsilateral lumbar DRGs of CCI-rats.

## Author Contributions

All authors designed the research. NK and AM performed the experimental work. NK analyzed the data and wrote the manuscript and prepared the figures. All authors reviewed, edited, and approved the final version of the manuscript.

## Conflict of Interest Statement

MS is an inventor on a UQ patent for the use of AT2 receptor antagonists in neuropathic pain that was commercialized by the UQ spin-out company, Spinifex Pharmaceuticals Pty Ltd., and that was acquired by Novartis in mid-2015. The other authors declare that the research was conducted in the absence of any commercial or financial relationships that could be construed as a potential conflict of interest.

## Supplementary Material

**FIGURE S1 |** Specificity of primary antibodies used for targeting the AT_2_ receptor. **(A)** Immunocytochemistry showed that the antibodies (rabbit polyclonal anti-AT_2_ receptor antibody; ab19134 and rabbit monoclonal anti-AT_2_ antibody, ab92445; Abcam) specifically labeled HEK cells expressing the AT_2_ but not the AT_1_ receptor or the non-transfected HEK-cells (control group). **(B)** Lane 1 shows the standard ladder whereas lanes 2–3 demonstrate that the AT_2_ receptor antibody, ab92445, specifically labeled HEK cells expressing the AT_2_ but not the AT_1_ receptor **(A)** Lane 4 shows that the HEK-AT_2_ cells treated with tunicamycin (TNM) at 1 μg mL^-1^ for 48 h expressed de-glycosylated forms of the receptor. Specifically, there were bands at ∼50 kDa similar to the band from DRG tissues, and at ∼90 kDa consistent with N-glycosylation of this receptor. Scale bars represent 25 μm. HEK 293, human embryonic kidney 293 cells; kDa, kiloDaltons.
