# Supplementary figures and images for: Attenuation of the Infiltration of Angiotensin II Expressing CD3+ T-Cells and the Modulation of Nerve Growth Factor in Lumbar Dorsal Root Ganglia – A Possible Mechanism Underpinning Analgesia Produced by EMA300, An Angiotensin II Type 2 (AT2) Receptor Antagonist
Source: Front Mol Neurosci. 2017 Nov 21;10:389. doi: 10.3389/fnmol.2017.00389 (PMC5696600; doi:10.3389/fnmol.2017.00389)

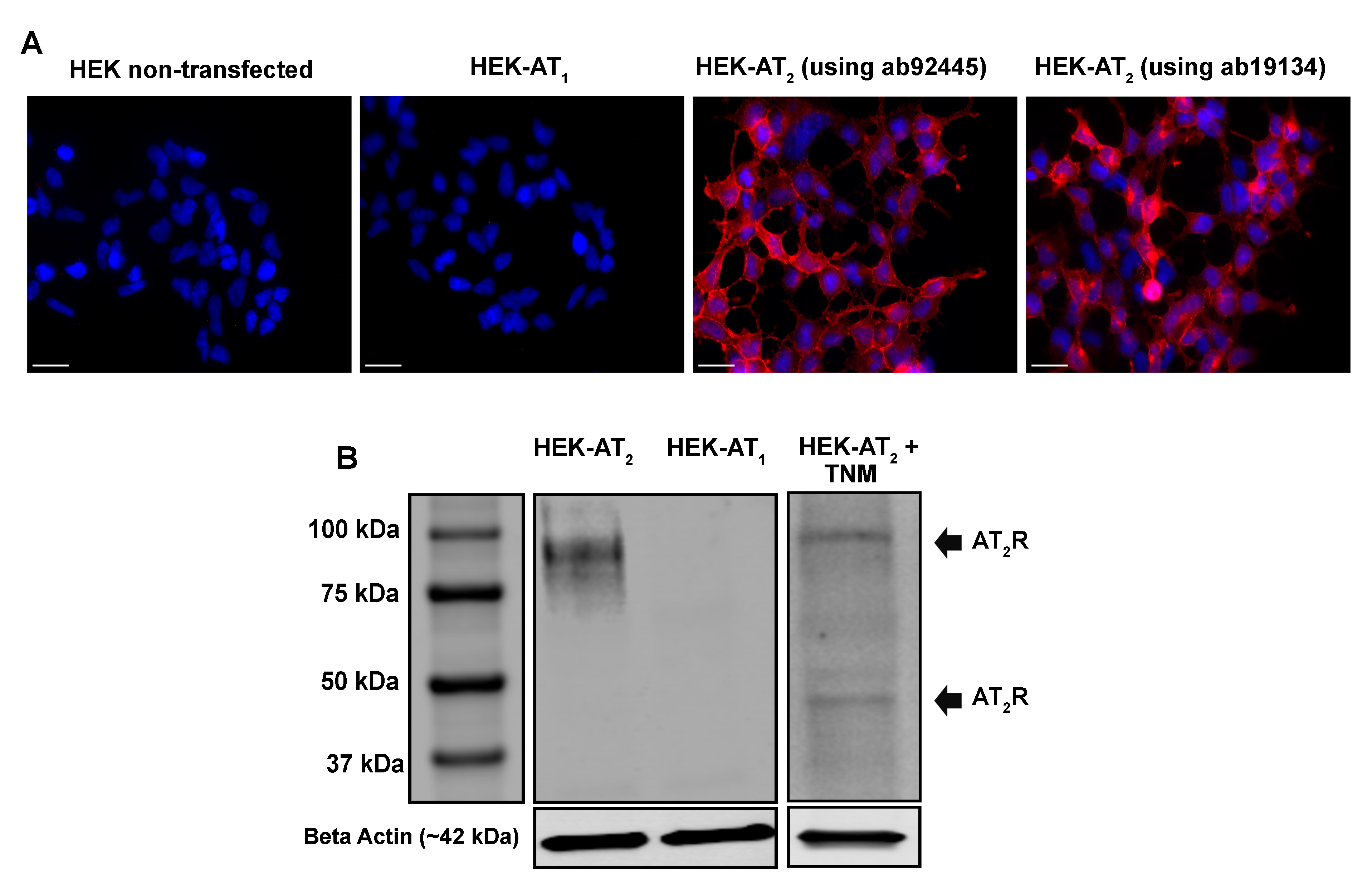

Supplement: Supplementary file 1 [file Image_1.TIF]
